# Brain morphologic abnormalities in migraine patients: an observational study

**DOI:** 10.1186/s10194-020-01109-2

**Published:** 2020-04-25

**Authors:** Lilla Bonanno, Viviana Lo Buono, Simona De Salvo, Claudio Ruvolo, Viviana Torre, Placido Bramanti, Silvia Marino, Francesco Corallo

**Affiliations:** IRCCS Centro Neurolesi “Bonino-Pulejo”, S.S. 113, Via Palermo, C. da Casazza, 98124 Messina, Italy

**Keywords:** Aura, Magnetic resonance imaging, Migraine, No Aura, Voxel-based Morphometry

## Abstract

**Background:**

Migraine is a common neurological disorder characterized by a complex physiopathology. We assessed brain morphologic differences in migraine and the possible pathogenetic mechanism underlying this disease.

**Methods:**

We analyzed brain morphologic images of migraine patients, 14 with aura (MwA) [the mean (SD) age was 42.36 (2.95) years (range, 37–47)] and 14 without aura (MwoA) [the mean (SD) age was 43.5 (3.25) years (range, 39–50)] during episodic attack compared with health subjects balanced (HS) [the mean (SD) age was 42.5 (5.17) years (range, 34–51)]. All subjects underwent a Magnetic Resonance Imaging (MRI) examination with a scanner operating at 3.0 T and voxel based morphometry (VBM) approach was used to examine the gray matter volume (GMV). The statistical analysis to compare clinicl characteristics was performed using unpaired t-test an one-way Anova. Results: Total cerebral GMV showed a significant difference between MwA and HS (*p* = 0.02), and between MwoA and HS (*p* = 0.003). In addition, not significative differences were found between MwA and MwoA groups (*p* = 0.17). We found three clusters of regions which showed significant GMV reduction in MwA compared with MwoA. MwA subjects showed a less of GMV in 4 clusters if compared with HS, and MwoA subjects showed a less of GMV in 3 clusters if compared with HS. We observed that MwA and MwoA patients had a significant reduction of GMV in the frontal and temporal lobe and the cerebellum, if compared to HS. The bilateral fusiform gyrus and the cingulate gyrus were increase in MwoA patients compared with HS.

**Conclusion:**

Our findings could provide a approach to understand possible differences in the pathogenesis of two type of migraine.

## Introduction

Magnetic resonance imaging (MRI) is an instrumental method which provides a non-invasive observation of brain changes. Voxel-based morphometry (VBM) is an automated technique using to analize the MRI images [1] and to identify changes in brain anatomy. It is characterized by high regional specificity and it does no require preventively the definition of a particular region of interest (ROI) [2]. VBM is largely used because it is relatively easy to use [3].

A recent VBM study, in which regional volumes based voxel-wise on were compared between patients with migraine and controls, detected structural differences in brain tissue composition of migraine patients [4]. Migraine is a common neurological disorder, characterized by recurrent unilateral pain attacks associated with nausea and other neurovegetative symptoms of moderate or severe intensity, whose physiopathology is complex [5]. Up to one third of migraineurs with aura have visual symptoms followed by motor somatosensory symptoms during attacks [6]. Literature data reported an association between migraine and morphologic brain alterations [7]. The aim of this study is to applie VBM approach in migraineurs patients with aura (MwA) and without aura (MwoA) in order to analyze their brain morphologic differences and to evaluate a possible pathogenetic mechanism underlying these two types of migraine.

## Methods

### Subjects

Twenty-eight migraine patients (14 MwA and 14 MwoA), according to International Headache Society criteria (Headache Classification Committee of the International Headache, 2013), and 14 sex and age matched healthy subjects (HS) were recruited. MwA group composed of 14 female patients with aura attacks of visual area. MwoA group composed of 14 female patients without aura attacks. Demographic and clinical characteristics showed in Table 1. The protocol was approved by the Local Ethics Committee of IRCCS Centro Neurolesi Bonino-Pulejo of Messina (Italy) and conducted according to the Declaration of Helsinki. Informed consent was obtained from all participants included in the study. The exclusion criteria were: 1) other types of headache; 2) vascular disease or trauma; 3) history of major psychiatric disorders; 4) presence of metabolic disorders; 5) aura greater than 70 min.

**Table 1:** Socio-demographic and clinical characteristics of three groups (% frequency)

	Migraine without aura (Mean±SD)	Migraine with aura (Mean±SD)	Health Control (Mean±SD)	p
N. patients	14	14	14	
Age	43.5±3.25	42.36±2.95	42.5±5.17	0.70^±^
Education	12.96±3.49	13.45±3.28	13.46±3.75	0.91^±^
Disease Duration (year)	6.78±3.66	5.21±1.31	–	0.14^¥^
Attacks per year	22.75±10.03	29.83±11.9	–	0.10^¥^
Frequency of attacks (mounth)	1.89±1.18	2.48±1.40	–	0.24^¥^
Duration of headache attacks (hours)	2.5±1.2	3.3±1.9	–	0.19^¥^
VBM analysis				
Grey Matter	793.83±47.10	814.73±29.10	850.98±46.11	0.003^*±^

^*^p < 0.05

^¥^unpaired t-test

^±^one-way Anova

*SD* Standard Deviation, *HAM-A* Hamilton Rating Scale for Anxiety, *BDI-II* Beck Depression Inventory

The patients were in treatment with analgesics (18/28), triptans (4/28) and combination of analgesics (2/28). We did not find cognitive impairment in our patients. All patient underwent a MRI examination with a scanner operating at 3.0 T (Achieva, Philips Healthcare, Best, The Netherlands), by using a 32-channel SENSE head coil. MRI scans were performed in the interictal phase at least 3 days after migraine attack. For each subject, T1 [TR = 8 ms, TE = 4 ms, slice thickness/gap = 1/0 mm, number of slices = 173, field of view 240 mm] was acquired.

### Voxel-based Morphometry method

We used VBM approach to examine the gray matter. The VBM is based on 3 basic preprocessing steps following by statistical analysis. These steps are: tissue classification, spatial normalization and spatial smoothing [2].

The local tissue morphology was maintained performing a modulation/correction for volume changes on segmented brain images. The latter were also levelled off with an isotropic 12 mm FWHM Gaussian kernel. Afterwards, the last step consisted in the esteem of global GM and WM volumes and total intracranial volume (TIV) by using segmented images in native space.

### Data processing and analysis

Image data processing was performed with SPM12 (www.fil.ion.ucl.ac.uk). We considered GM tissue to calculate the GM tissue volume (GMV) and TIV in the native space. Subsequently, we used the affine registration algorithm to record all the native-space tissue segments to the standard Montreal Neurological Institute template (included in SPM12). The use of the exponentiated lie algebra toolbox (DARTEL) to all participants’ GM and WM was necessary to refine the inter-subject registration via the application of the diffeomorphic anatomical registration. In the last step of DARTEL, we used a non-linear approach to modulate the GM tissues, in order to compare the relative GMV tailored for individual brain size. In addition, we performed the spatial normalization [8] to estimate the Jacobian determinant that was used to modulate the voxel values in the tissue maps. Moreover, an assessment of the GM tissue homogeneity was needed. For this reason, we performed a quality check with a CAT12 toolbox after preprocessing pipeline. Finally, a Gaussian filter with 8 mm of FWHM was used to fit the GM tissue segments modulated and normalized for each subject.

### Statistical analysis

The VBM analysis was carried out using the CAT12 toolbox in MatLab (www.mathworks.com). The first step consisted to perform a 2-sample t-test, with age, attacks per year, frequency of attack, duration of headache attacks and TIV as covariates, to compare the GMV between patients and HCs. Statistical parametric maps were generated after family-wise error (FWE) correction for multiple voxel-wise comparison. These maps were created using an initial threshold *p* < 0.001 and estimated at peak statistical significance level for *p* < 0.05. The extent threshold was set at 100 voxels.

## Results

**Fig. 1:**
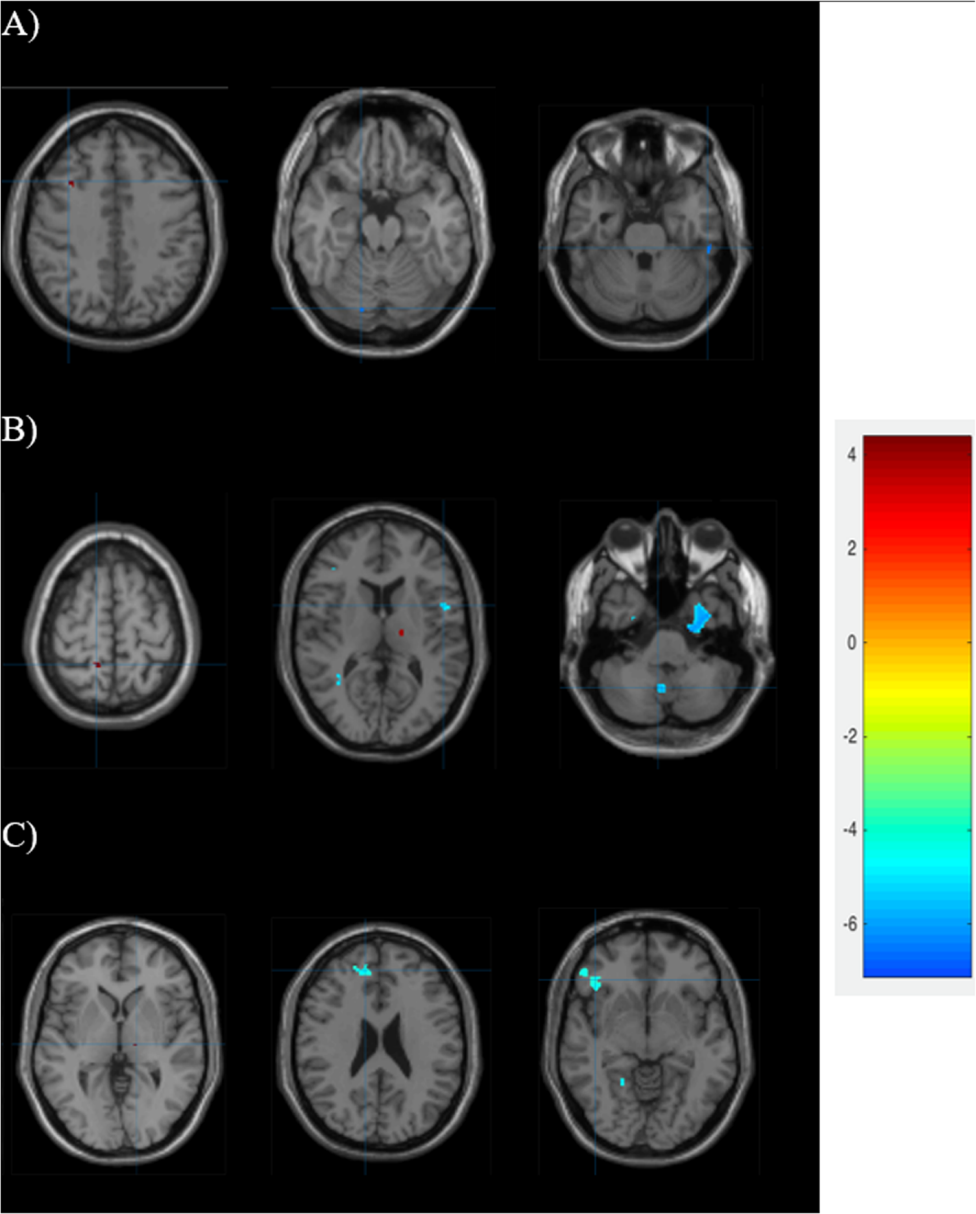
Gray matter volume (GMV) changes. A) GMV of migraine with aura compared with migraine without aura. B) GMV of migraine with aura compared with health control. C) GMV of migraine without aura compared with health control. Statistical parametric maps show gray matter volume alterations with a threshold of *P* < 0.001 uncorrected superimposed on a standard T1 image. The color bar reflects t values (red/yellow = increased volume, blue = decreased volume). MwA = Migraine with Aura; MwoA = Migraine without Aura; HS = Heath Subjects

All participants completed the study. The total cerebral GMV showed a significant difference between MwA and HS (*p* = 0.02), and between MwoA and HS (*p* = 0.003). In addition, not significative differences were found between MwA and MwoA groups (*p* = 0.17) (Table 1). We corrected the analysis for multiple comparisons (*p* < 0.05 family-wise error corrected), and we detected the regions with significant GM changes between MwA and MwoA groups. At *p* < 0.05, we found 3 clusters of regions that showed significant GMV reductions in MwA if compared with MwoA and more GMV in the right frontal lobe (Fig. 1 and Table 2). MwA group showed less GMV in 4 clusters (right cerebellum, left postcentral and precentral gyrus, right inferior frontal gyrus, left Broadman area 20–22 and left lingual gyrus), and more GMV in right superior parietal gyrus and left thalamus (Fig. 1 and Table 2) if compared to HS. Finally, MwoA subjects showed less GMV in 3 clusters (bilateral cerebellum, left cerebellum crus I, left superior/medial and right inferior/middle frontal gyrus, right superior frontal gyrus, left fusiform gyrus, left Broadman area 20, right parahippocampal gyrus and insula) and more GMV in right thalamus (Fig. 1 and Table 2) if compared to HS.

**Table 2:** Significant regions with less or more grey matter volume in migraine patients compared with controls

	Location	Coordinates (mm)			Peak Z	Corrected p
		x	y	z		
MwA vs MwoA	L Inferior Temporal	−52	−34	− 27	− 3.76	0.04
	R Frontal lobe	33	18	40	3.83	0.04
	R Cerebellum	16	−82	−20	−3.51	0.04
MwA vs HS	R Cerebellum	2	−64	− 57	−5.14	0.01
	L Postcentral gyrus	−26	30	74	−4.87	0.03
	R inferior frontal gyrus	39	34	9	−3.51	0.01
	L Brodmann area 22	−58	8	−2	−4.30	0.04
	L lingual gyrus	−15	−46	−4	−3.52	0.03
	R superior parietal gyrus	15	−45	63	4.12	0.02
	L Thalamus	−14	−15	10	3.67	0.01
MwoA vs HS	2003Cerebellum	9	−60	− 54	−7.03	0.03
	L Cerebellum crus 1	−45	−64	−24	−4.27	0.02
	L superior/medial frontal gyrus	−10	38	39	−4.54	0.004
	R Inferior/middle frontal gyrus	40	30	−6	−4.74	0.01
	R superior frontal gyrus	32	57	26	−4.29	0.001
	L fusiform gyrus	−22	−62	−15	−4.31	0.001
	L Brodmann area 20	−34	−20	−33	−5.25	0.02
	R parahippocampal gyrus	24	−52	−3	−5.14	0.04
	R Insula	40	30	−6	−4.74	0.03
	R Thalamus	15	−18	3	4.33	0.04

Legend: *MwA* Migraine with Aura, *MwoA* Migraine without Aura, *HS* Heath Subjects, *R* Right, *L* Left, *BA* Brodmann’s area

## Discussion

Neuroimaging developments have provided highly sensitive and non invasive approaches to investigate the neural mechanisms of brain alterations associated with several disorders. Although advances in migraine research contributed to improve the disease understanding, the use of advanced MRI techniques allowed the accurate investigation of migraine patients. Migraine is not only relate to pain occurring intermittently or constantly, but a process that over time affects the brain acting on a predisposed brain (genetic) and modifining it the function or morphology. Several fMRI studies revealed abnormalities of resting state functional connectivity in pain network involved in migraine pathophysiology [6, 9, 10]. Abnormalities have been reported in multiple brain areas as evidenced by VBM. It is an ongoing matter of debate whether the changes are cause or consequence of migraine, but in many VBM studies the changes correlated with disease duration argues in favor of the latter. The exact underlying mechanisms, leading to alterations in grey matter density as evidenced by VBM, are not clear. These alterations may reflect modifications of dendritic complexity or changes in the numbers of synapses or simply in water content. These changes may be an index of the disorder, its progression or an effective therapy. Migraine patients showed significant GM abnormalities of several brain regions involved in central pain processing [11, 12]. In particular, VBM data established that the GMV was decreased in the anterior cingulate cortex, insula, amygdala, parietal operculum, middle and inferior frontal gyrus [11]. In addition, regions with less grey matter density are located in bilateral insula, motor premotor, prefrontal, cingulate cortex, right posterior parietal cortex and orbitofrontal cortex [13].

In this study, we applied the VBM approach to MwA and MwoA patients and HS. We observed that MwA and MwoA subjects had a significant reduction of GMV compared to HS in cerebellum, and frontal and temporal lobe. Our previous study [6] analyzed the resting state findings in the same patient sample and we found an hyperactivity increase of cortical activity in bilateral fusiform and cingulate gyrus of MwoA subjects compared with controls. In this study, the VBM approach showed a reduction in the volumes of same cerebral areas. Although the volume of bilateral fusiform and cingulate gyrus is descreased, the hyperactivity of cortical activity could be ascribed to the fusiform gyrus that seems to be hyperactive in migraineurs for it involvment in the cognitive pain treatment, while the cingulate gyrus is involved in the transformation process of migraine from “an episodic” to “a chronic brain disorder” [14]. Fusiform gyrus is involved in nociception/antinociception and neurocognitive aspects of pain processing. In idiopathic or primary headaches, including migraine, tension-type headaches, and cluster headaches, the accepted view is that these conditions are due to abnormal brain function that occurs with normal brain structure. A decrease in GMV suggest that the central reorganization processes in chronic pain syndromes may involve degeneration of anti-nociceptive brain areas.

In addition, the cerebellum of migraineurs and controls differs structurally. In a study of Mehnert [15] the GMV and the neuronal activity, in response to trigeminal pain, increased in posterior part of the cerebellum (crus). Migraine patients had also a connectivity decrease in the thalamus and higher cortical areas, suggesting a less inhibitory involvement of migraine cerebellum on trigeminal nociception. The frontal cortex is the area associated with cerebral abnormalities in migraine patients [16, 17]. Previous studies suggested that the medial prefrontal cortex could be involved in mediating the attenuation of pain perception by a cognitive control mechanisms [18, 19], associated with pain modulation [20, 21]. Schmitz et al. [22] reported that migraine patients had a less gray matter density in the medial prefrontal cortex correlated significantly with a slower response time to the set-shifting task.

In coherence with previous VBM findings, [11, 12, 17] our results corroborate with the study of Kim et al. [4], that found a less volume of insula bilaterally, motor/premotor, prefrontal and cingulate cortex, right posterior parietal cortex, and orbitofrontal cortex. Moreover, Jin et al. [9] showed a less GMV in several brain regions involved in pain processing, such as left medial prefrontal cortex, cingulate, right occipital lobe, cerebellum, and brainstem. The results obtained affirm that migraine patients have a less GMV in the precentral gyrus as well as in the post-central gyrus and temporal lobe.

In literature, the possible mechanisms underlying the reduction of grey matter in migraine are currently unknown. The observed decrease in grey matter may reflect tissue shrinkage (changes in extracellular space and microvascular volume) as well as more complex processes as neurodegeneration. Therefore, there are several possible explanations for the observed abnormalities in our patients. Variations in gray matter may result from repeated ischaemia caused by blood flow both during migraine attacks and in the interictal phase. In contrast, the reduction of gray matter may be a consequence of migraine specific neurotoxic mechanisms. It has been hypothesized that migraine is associated with a state of neuronal hyperexcitability, involving over-activity of the aminoacid exciters glutamate and aspartate. VBM analysis shown that migraineurs present a significant reduction in the gray matter of different brain areas involving to the pain activation network [6].

## Conclusion

The VBM approach is an important and useful tool to assess brain morphologic changes in neurological disorders, such as migraine. The different results reported by aforementioned studies could be attributed, in part, to the use of different MRI scanners (3 T vs. 1.5 T) [23, 24]. In fact, different scanners may led to the different approaches for GM segmentation and to detect morphologic abnormalities of different types. In addition, migraine is a heterogeneous disorder, whereby it is difficult to obtain a phenotypically homogeneous group.

Although we investigated a small sample of patients, our results could provide a new instrumental approach useful to understand the pathogenesis of MwA and MwoA.

## Data Availability

The datasets generated and/or analyzed during the current study are not publicly available due to the Local Ethics Committee but are available from the corresponding author on reasonable request.

## Abbreviations

MwA: Migraine with aura
MwoA: Migraine without aura
HS: Health subjects
MRI: Magnetic Resonance Imaging
GMV: Gray matter volume
NBV: Normalized brain volume
ROI: Region of interest
TIV: Total intracranial volume
FWE: Family-wise error
